# The College and Nurses' Hours

**Published:** 1920-04-10

**Authors:** 


### THE MATRONS' AND SISTERS' DEPARTMENT.

### THE COLLEGE AND NURSES' HOURS.

No measure has ever come before the House of Commons likely to affect nurses' interests more closely than the Bill to regulate workers' hours of employment, which is shortly to be introduced. As our readers know, it has long been under discussion in the circles most interested in its provisions. Its promotors are most anxious to have the considered opinion of every class of worker, with a view to making its clauses fair and workable. And it is certain that special clauses applicable to nursing work must be devised if this large body of over-worked women is to share the relief afforded by the Bill to other women workers.

It is difficult to understand how the nursing profession could have been approached with any reasonable hope of obtaining an answer of a truly representative character had it not been for the existence of the College of Nursing. Mindful of its responsibilities towards the profession and towards the public, this body some time ago formed a Parliamentary Committee with a view to watching and reporting on any measures brought before Parliament which might affect the interests of nurses. The machinery was therefore ready when the hour came and could be set in motion without delay or the makeshift policy of appointing a committee in a hurry. The Ministry of Labour has now appealed to the College of Nursing for a scheme of nurses' hours which can be put into practice under the Hours of Employment Bill, without dislocating the nursing service. A scheme has already been drawn up and it has been under consideration ever since the Bill was first drafted and is now nearly ready. The Council have, however, postponed their final decision as to the numerous details which require thinking out, the many claims which have to be adjusted, until each local centre has deliberated upon the draft scheme and forwarded its observations thereon to the College headquarters. The value of this patient investigation into the new principle which is to be introduced into nursing cannot be over estimated. A scheme drawn up at headquarters might be efficient. It could hardly be accepted as final unless thoroughly ventilated by the representatives in every part of the country of those on whom will fall the onus of making it work when it becomes law.

It is matter for profound satisfaction that nurses should be admitted to the benefits which it is hoped to confer on workers, male and female, through the Hours of Employment Bill. Because of the unceasing claims of the sick by night and by day, it has been too generally held for true that nurses' hours must know no limitations but those imposed by nature. Their unselfish devotion to duty, the appeal of their own hearts, have often been brutally turned against them to their undoing. The extent to which they are still, in numerous institutions and in private work, tortured for lack of leisure is notorious. It is the good employer who is best fitted to lead the way in demonstrating how far the eight-hour day, the 48-hour week, or the 96-hour fortnight may be made compatible with the nurse's duty to the sick. Few nurses who have not borne the onus of directing the nursing administration in a modern, well-equipped hospital could be trusted to frame a scheme under which nurses can be rescued from over-long hours on duty without producing worse evils.

Unquestionably, from the nursing point of view, this is the most important measure ever devised. People may still ask of State registration, "What is the good of it?" but no one will ever pause to ask this stupid question about the Hours of Employment Bill. For the burning question of the moment in the nursing profession is not salaries, but hours. Once that is settled, once the abuses lurking in a thousand unreformed corners of this country are swept away with a strong Parliamentary broom (and we fear no other bristles are strong enough for the task), there will be a great revival of public interest in nursing. Hesitating mothers will be reassured. Doubting heads of schools and colleges will lend an attentive ear to the call of the training-schools. The active, strong, and eager probationers whom every matron welcomes, will no longer be warned against hospital life by their friends who happen to have tried it.

What will the future hold for the nurse who is no longer too tired to think when she comes off duty? How will the patient fare under nurses no longer too hurried and harried to let their sympathies have play? These are things hidden in the future. We plant in faith and we know that the fruit must be good. We know that new conditions mean new dangers, but we are confident that there is wisdom enough among us to avert them.

